# Post-exercise pulsatility index indicates treatment effects in peripheral arterial occlusive disease (PAOD)

**DOI:** 10.1007/s00508-021-01818-x

**Published:** 2021-03-11

**Authors:** Andrej Udelnow, Maria Hawemann, Ivo Buschmann, Frank Meyer, Zuhir Halloul

**Affiliations:** 1 Dept. of Endovascular and Vascular Surgery/Phlebology, Brandenburg Medical School Theodor Fontane, Dessau Municipal Hospital, Dessau, Germany; 2 Division of Vascular Surgery, Dept. of General, Abdominal, Vascular and Transplant Surgery, Magdeburg University Hospital, Magdeburg, Germany; 3 Dept. of Angiology, Brandenburg Medical School Theodor Fontane, Brandenburg Municipal Hospital, Brandenburg, Germany; 4 Dept. of General, Abdominal, Vascular and Transplant Surgery, Magdeburg University Hospital, Magdeburg, Germany

**Keywords:** Hemodynamic parameter, Peak systolic velocity (PSV), End-diastolic velocity (EDV), Minimal diastolic velocity (MD), Time-averaged maximal velocities (TAMAX), Resistance index (RI), Resting measurement, Standard exercise test, Reintervention-free survival

## Abstract

**Background:**

Hypothesis: Post-exercise measurements better discriminate PAOD-patients from healthy persons and they more sensitively detect hemodynamic improvements after treatment procedures than resting measurements.

**Methods:**

A total of 19 healthy volunteers and 23 consecutive PAOD-patients underwent measurements of peak systolic velocity (PSV), end-diastolic velocity (EDV), minimal diastolic velocity (MDV), time-averaged maximum velocities (TAMAX), resistance index (RI) and pulsatility index (PI) before and after a standard exercise test (at 1, 2, 3, 4 and 5 min) before and after treatment (incl. epidemiological data, PAOD risk factors and comorbidities).

**Results:**

In resting values, healthy persons and PAOD-patients did not differ significantly in any of the hemodynamic parameters. PSV increased after treatment in PAOD-patients by 5 cm/s (paired t‑test, *p*: 0.025); however, when the amplitude of autoregulatory changes related to the resting values were calculated, PAOD-patients showed clearly less hemodynamic changes after exercise than healthy persons (*p*: 0.04; 0.002; <0.001 for PSV, TAMAX and PI, resp.). The time course after exercise was compared by repeated measures of ANOVA. Healthy persons differed significantly in PI, RI and PSV from PAOD patients before and after treatment (*p*<0.001 each). The PAOD-patients revealed a significantly improved PI after treatment (*p*: 0.042). The only factor contributing significantly to PI independently from grouping was direct arterial vascularization as compared to discontinuous effects by an obstructed arterial tree.

**Conclusion:**

Healthy persons cannot be well differentiated from PAOD-patients solely by hemodynamics at rest but by characteristic changes after standard exercise. Treatment effects are reflected by higher PI-values after exercise.

## Introduction

Vascular medicine depends heavily on non-invasive measuring hemodynamic parameters, such as ankle-brachial index (ABI), peak systolic velocity (PSV), end diastolic velocity (EDV), minimum diastolic velocity (MD) and time averaged maximal velocity (TAMAX). Calculated values, such as the resistance index (RI) and the pulsatility index (PI) are also used commonly for interindividual and intraindividual comparisons. Sceptics argue that several error sources, such as media sclerosis, heart insufficiency, the Doppler probe positioning and other user-dependent factors may influence the correctness of the results [1]. The evidence supposing a predictive role for prognosis of treatment effects is sparse at best [2]. Even the contemporary peripheral arterial obstructive disease (PAOD) classification systems, such as Inter-Society Consensus for the Management of Peripheral Arterial Disease (TASCII), prefer clinical over objective hemodynamic criteria (ancle brachial index [ABI]) [3]. On the other hand, crural ulcers may have different etiologies in the presence of PAOD, and the lack of objective evidence-based criteria to differentiate PAOD from other etiologies makes it difficult to find an appropriate treatment even for these routine cases. Other non-invasive methods and parameters, such as transcutaneous partial oxygen pressure measurement, “transkutane Sauerstoffpartialdruckmessung” (tcpO_2_), contrast-enhanced ultrasonography (CEUS) and toe pressure measurements try to circumvent the methodological artifacts of Doppler ultrasonography by quantifying the perfusion or oxygenation at the periphery but again, available evidence is not sufficient to recommend these methods for standard diagnostics [3].

Are these hemodynamic parameters able to reflect what they are believed to, namely to quantify the real capacity of the vascular system to transport blood to the tissues and to satisfy the changing needs in oxygen? It is known that the vascular autoregulation includes constriction of the precapillary sphincters at rest and their dilatation at exercise in order to redistribute the blood between viscera and extremities. The typical triphasic pulse wave form converts to monophasic flow for some minutes after exercise in the crural arteries in healthy persons indicating a physiologic lowering of the peripheral resistance; however, the poststenotic or postocclusion flow is typically also monophasic in PAOD patients already in resting states, pointing to altered autoregulation. Logically, beside the different pulse wave morphology at Doppler ultrasonography, the blood volume per minute should be about the same at rest in healthy persons and PAOD patients (at least in Fontaine-II stage), and the functional reserve may differ between them (when measuring the blood volume per minute after exercise). Furthermore, the hemodynamic parameters and the pulse wave form may not differ at rest in patients before and after successful treatment, particularly in those patients whose crural arteries are fed via collaterals, but only after exercise. Starting from this consideration, the aim of the present investigation was to compare the Doppler ultrasonography-based hemodynamic parameters mentioned above before and after exercise in healthy persons and PAOD patients (before and after a treatment procedure). The hypothesis was that the change or course of the post-exercise hemodynamics would differentiate between healthy persons and PAOD patients and between successful and frustrating treatment while the resting state measurements would not.

## Patients and methods

### Study design

The investigation was performed as a prospective non-randomized, non-invasive diagnostic single-center study in a consecutive patient cohort consisting of two parts: a comparison of two independent cohorts (healthy volunteers and PAOD patients) and an intra-cohort comparison of the states before and after treatment in PAOD patients.

### Patients and volunteers

In this study 19 healthy volunteers and 23 PAOD patients hospitalized in the Division of Vascular Surgery of the Magdeburg University Hospital, Germany, were included from October 2015 until June 2016 based on informed consent.

### Exercise test

Healthy volunteers were examined by duplex ultrasonography (General Electric, Logiq E9, 9 MHz linear probe, Freiburg, Germany) and the hemodynamic parameters of the right anterior tibial artery (ATA) were measured or calculated by the integrated software after 5min of resting in supine position at the medial third of the lower leg (PSV, EDV, MD, TAMAX, RI, PI). Then, they were asked to perform dorsal flection of the feet in upright position 60 times within 1min or until claudication occurred. The ultrasound examination was repeated 1, 2, 3, 4 and 5 min after the test. Patients underwent the same test in hospital before and after the treatment; however, when they were not able to take part in the test in upright position, they were allowed to perform the test supine with slightly elevated legs. Depending on the vascular state, one of the perfused crural vessels was examined with preference for the ATA.

### Clinical data and follow-up

Besides the assessed Doppler parameters, data from the medical history, such as age, weight, height, sex, predisposing factors, such as smoking, diabetes, hyperlipoproteinemia, thrombophilia, genetic predisposition and cardiovascular risk factors were asked. Furthermore, the lower extremity vessels were assessed by the clinical routine diagnostics (digital subtraction angiography [DSA], magnet resonance angiography [MRA]) and whether the examined artery was directly perfused or via collaterals was documented. The patients were followed-up after discharge from hospital until December 2016, and the censored survival, major amputation and reintervention was assessed.

### Ethics

According to the legal requirements of the State of Sachsen-Anhalt in Germany, for the non-invasive study using data of the routine diagnostics, approval by the local ethics committee was not necessary; however, the data safety guidelines of the Magdeburg University and the State of Sachsen-Anhalt were strictly followed.

### Statistics

For statistical calculations, the R software was used [4], ANOVA was performed using the package NLME [5], the Shapiro test with the mvnormtest package [6], survival analysis with the survival package [7]. Differences were considered significant when *p* < 0.05 and a tendency was defined as *p*: 0.05 … 0.1.

## Results

### Resting hemodynamics in healthy persons and PAOD patients

The general characteristics of the cohorts were compared in the factorial analysis subsection.

Table 1 shows the mean values for each parameter. It is obvious that the hemodynamic parameters did not significantly differ between the healthy cohort and the PAOD cohort. Only the RI showed a tendency towards lower values in PAOD patients because almost all healthy persons had triphasic pulse wave forms at rest, which is equal to a RI of 1. When the values of PAOD patients before and after treatment were compared, the PSV was slightly but significantly elevated after treatment, which points to better perfusion after treatment. An analogous tendency was observed for MD and RI. The tcpO_2_ quantifying the tissue oxygenation was not measured in healthy persons. The comparison between before and after treatment in PAOD patients did not result in a significant difference. Summarizing, we can state that healthy persons and PAOD patients differed only by RI while a significant (but minimal) treatment effect in PAOD patients could only be observed for the PSV.

**Table 1:** Comparison of hemodynamic parameters means in crural arteries in resting state

Parameter	Healthy cohort	Patient cohort before treatment	*P*^a^	Patient cohort after treatment	*P*^b^
PSV	55	46	0.3	50	*0.025*^c^
ED	0.4	11	0.1	7	0.17
TAMAX	13	19	0.3	18	0.98
MD	0	11	0.3	8	0.07^d^
PI	4.7	2.8	0.1	3.0	0.5
RI	1	0.75	0.057^d^	0.86	0.08^d^
tcpO_2_	NA	49	NA	56	0.36

The comparisons between the pretreatment and posttreatment states used the same arteries, while the comparisons between healthy persons and PAOD patients did not in all cases because of arteriosclerotic obstructions in PAOD patients

*NA* not assessed, *PSV* peak systolic velocity, *ED* end-diastolic velocity, *MD* minimal diastolic velocity, *TAMAX* time-averaged maximal velocities, *RI* resistance index, *PI* pulsatility index, *tcp02* transcutaneous partial oxygen pressure measurement (“transkutane Sauerstoffpartialdruckmessung”)

^a^non-paired, two sided *t*-test between healthy cohort and PAOD cohort before treatment

^b^paired, two-sided *t*-test between the PAOD patients before and after treatment

^c^significant difference

^d^tendency

### Amplitudes of autoregulation in hemodynamic parameters after exercise test

**Fig. 1:**
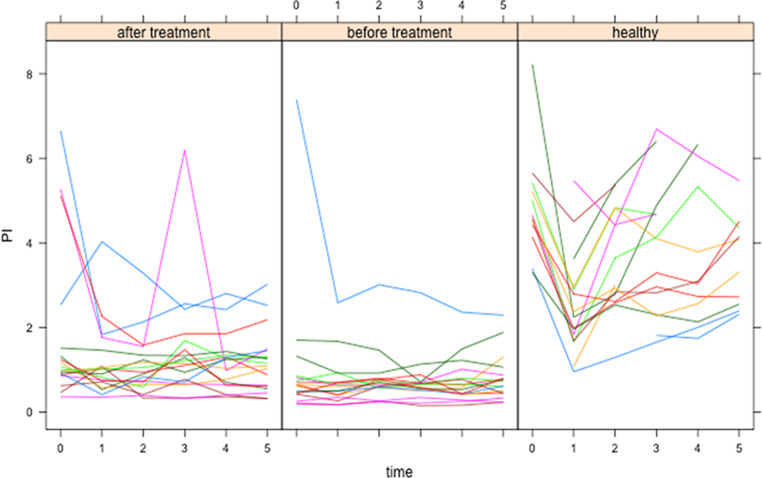
Individual courses of the PI before (time = 0 min) and after exercise in healthy persons and PAOD patients (before and after treatment). The initial drop 1 min after exercise in healthy persons and some of the PAOD patients can be explained by physiological peripheral dilatation of arterioles. The lack of this drop is characteristic for the disturbance of peripheral vegetative autoregulation after exercise

Both cohorts underwent a standardized exercise test and were measured thereafter every minute for 5 min. The individual courses of the PI after exercise are shown as examples of the hemodynamics in Fig. 1. While the physiological initial drop in PI, caused by peripheral dilatation of arterioles 1 min after exercise could be observed in all healthy persons, followed by rapid elevation back to the normal level, in PAOD patients before treatment, this course was present only in one case. In all other patients, the changes were delayed or diminished, and the values were in general lower than in healthy persons. After treatment, three patients showed an improved PI with almost restored physiological autoregulation. In order to quantify the autoregulation, we divided the minimal PI value measured from minute 1 up to minute 5 by the starting value before exercise. Following the physiological reactions for the parameters, the maximum values after exercise were taken for PSV and TAMAX and the minimum ones for RI, PI for calculation of these autoregulation ratios which were compared in Table 2. For EDV and MD, these ratios were not calculated because these parameters were zero for most healthy people.

**Table 2:** Comparisons of autoregulation ratios before and after exercise hemodynamics

Parameter	Healthy cohort	Patient cohort before treatment	*P*^a^	Patient cohort after treatment	*P*^b^
PSVmax/PSV	1.6	1.2	*0.04*^c^	1.1	0.13
TAMAXmax/TAMAX	2.8	1.3	*0.002*^c^	1.7	0.13
PImin/PI	0.47	0.77	*<0.001*^c^	0.64	0.13
RImin/RI	0.82	0.77	0.374	0.79	0.33

The comparisons between the pre- and posttreatment state used the same arteries, while the comparisons between healthy persons and PAOD patients did not in all cases because of arteriosclerotic obstructions in PAOD patients. *PSV* peak systolic velocity, *ED* end-diastolic velocity, *MD* minimal diastolic velocity, *TAMAX* time-averaged maximal velocities, *RI* resistance index, *PI* pulsatility index, *tcp02* transcutaneous partial oxygen pressure measurement (“transkutane Sauerstoffpartialdruckmessung”)

^a^non-paired, two-sided *t*-test between healthy cohort and PAOD cohort before treatment

^b^paired, two-sided *t*-test between the PAOD patients before and after treatment

^c^significant difference

### Comparisons of the time courses after exercise by repeated measures ANOVA

It would be of value not only to discriminate healthy persons from PAOD patients but also treatment success from failure. Since autoregulation seems to remain disturbed after treatment but absolute values may differ, we used repeated measures ANOVA to assess the differences in the time course of hemodynamic parameters after exercise in healthy persons and PAOD patients before and after treatment. The results of the post hoc analyses are listed in Table 3.

**Table 3:** Post hoc analysis results for repeated measures ANOVA

Parameter	Healthy *vs*. PAOD before treatment		Healthy *vs*. PAOD after treatment		PAOD before *vs*. after treatment	
	Estimate	*p*	Estimate	*p*	Estimate	*p*
PI	2.47	*<0.001*^a^	2.10	*<0.001*^a^	−0.37	*0.042*^a^
RI	16.3	*<0.001*^a^	21.8	*<0.001*^a^	5.55	0.102
PSV	39.2	*<0.001*^a^	35.4	*<0.001*^a^	−3.81	0.261
TAMAX	3.58	0.30	2.48	0.58	−1.10	0.78
EDV	−4.25	0.061^b^	−1.44	0.82	3.10	0.12
MD	−6.09	*0.003*^a^	−3.2	0.20	2.90	0.15

The ANOVA was performed using the linear mixed effect model (lme)

Every model was compared to baseline by Akaike Information Criteria (AIC) and was significantly better (lower AIC). The Akaike information criterion (AIC) is an estimator of prediction error and thereby relative quality of statistical models for a given set of data. AIC estimates the relative amount of information lost by a given model: the less information a model loses, the higher the quality of that model [8]

*PSV* peak systolic velocity, *ED* end-diastolic velocity, *MD* minimal diastolic velocity, *TAMAX* time-averaged maximal velocities, *RI* resistance index, *PI* pulsatility index, *tcp02* transcutaneous partial oxygen pressure measurement (“transkutane Sauerstoffpartialdruckmessung”)

^a^significance

^b^tendency

### Influence of cofactors on PI

**Fig. 2:**
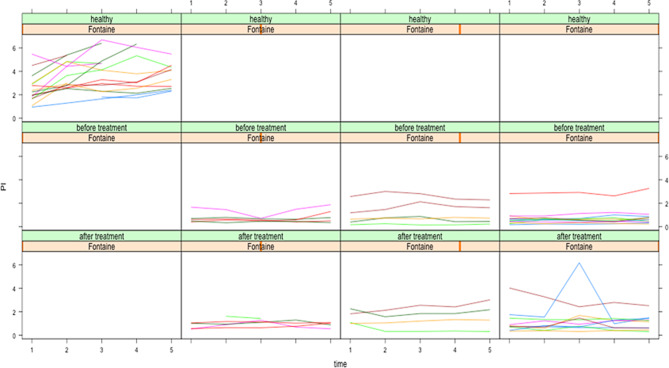
PI after exercise in patients with various Fontaine stages of PAOD and healthy persons (time in minutes). The left column contains Fontaine 0 (healthy) persons, the second stage IIb, the third stage III and the fourth stage IV by Fontaine classification. Furthermore, the first row is solely for the healthy persons without treatment while the other rows contain the patient cohort before (*second row*) and after (*third row*) treatment

Since Fig. 1 demonstrated a large range of the PI values in patients before and after treatment as well as in healthy persons, one of the further aims of the investigations was to analyze the univariate and multivariate influences of patient-related and disease-related cofactors on the PI. Fig. 2 shows the association between Fontaine stage, treatment and PI.

It is obvious that already in stage II of the Fontaine classification, the PIs were seriously lowered and invariable and did not change in further stages or following treatment.

**Fig. 3:**
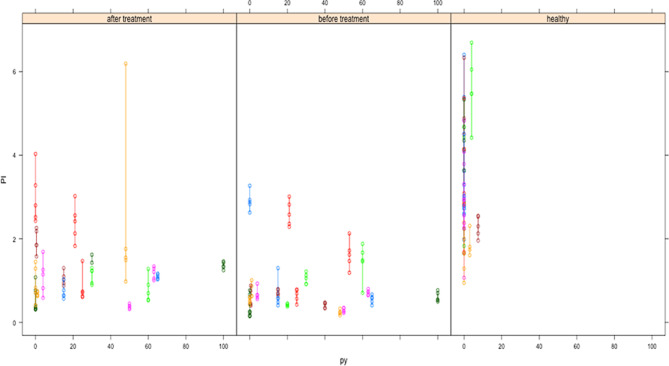
PI after exercise depending on the amount of py in the two cohorts. Each person/patient has a different line and color. It seems that there is an inverse statistical relationship—the higher the amount of py, the lower the PI

Since most of the PAOD patients are smokers, the influence of the amount of packyears (cigarette packs per day multiplied by years of smoking, py) was also investigated (see Fig. 3).

This univariate influence is highly significant as calculated by lme (statistical) package (*p*: 0.0025). Other factors, such as age, diabetes mellitus (DM), hyperlipoproteinemia (HLP), did not exhibit any univariate association with the PI. Since univariate statistics are not appropriate to enlighten causal relationships, a multiple regression analysis including grouping, Fontaine classification, py, DM and vascular connection (direct or via collaterals) was performed. The initial and the final models after regression are shown in Table 4.

**Table 4:** Multiple regression analysis of cofactors influencing the PI after exercise

Parameter	Initial model		Final model	
	Value	*p*	Value	*p*
Intercept	3.5	*0.0081*	3.3	*<0.0001*
Group: before therapy	−0.3	*0.0325*	−0.4	*0.0285*
Group: healthy	0.9	0.421	1.2	*0.0030*
Fontaine	−0.2	0.422	–	n. s.
Py	−0.006	0.37	–	n. s.
DM	0.4	0.26	–	n. s.
Direct vasc. preop	−1.02	0.08	−1.14	*0.0037*
Direct vasc. postop	0.03	0.94	–	n. s.

Grouping and pre-treatment direct vascular connection are independent factors with a significant impact

The other factors have been stepwise eliminated before by descending *p*-values

*Py* pack years, *DM* Diabetes mellitus, *vasc.* vascular, *preop.* preoperative(ly), *postop.* postoperative(ly), *n.* *s.* not significant

## Discussion and conclusion

One of the aims of the study was to assess whether a healthy cohort can be differentiated from a PAOD patient cohort in the resting state by the mean hemodynamic parameters. Furthermore, these were compared in the PAOD patient cohort before and after treatment.

As shown in Table 1, in particular representing “hemodynamics in resting subjects comparing healthy persons and PAOD patients”, in can be stated that based on these data it would be difficult to differentiate healthy persons from PAOD patients related solely to hemodynamic parameters of the crural arteries. Furthermore, the short-term treatment effects were not impressive with respect to the hemodynamic changes.

With respect to the “amplitudes of autoregulation in hemodynamic parameters after exercise test”, obviously healthy persons and PAOD patients can be identified much better by the autoregulation differences after exercise than by the resting values because the mean PSV, TAMAX and PI autoregulation ratios differed significantly between them. As would be expected from Fig. 1, the hemodynamic changes after exercise are more expressed in healthy persons pointing to intact autoregulation; however, when the ratios were compared in PAOD patients before and after treatment, no significant differences could be found. This can be explained by the fact that in most patients the autoregulation remained disturbed at the microvasculature level and an improvement is visible in only three patients Fig. 1.

As can be seen in the “Results” paragraph on “comparisons of the time courses after exercise by repeated-measures-ANOVA”, only the PI after exercise allows a treatment effect in the cohorts under investigation to be postulated, whereby the PI values were lower before treatment than after (negative estimate); however, as with PSV before exercise, this effect remained moderate.

Related to the “Results” paragraph entitled “influence of cofactors on PI” it can be summarized that:

- It seems that the hemodynamic changes characteristic for PAOD patients (lower perfusion parameters and diminished autoregulation after exercise) are present at an early stage of PAOD when there is still only claudication.
- Only sub-classifying patient into groups and direct vascular connection before treatment contributed significantly to the PI whereby the latter showed a negative influence, probably due to autoregulation after exercise.

Basically and in a general context, despite good concordance between Duplex ultrasonography and digital subtraction angiography (DSA) [9], Doppler and Duplex ultrasonography have not yet fulfilled their promises to predict the clinical course in PAOD and to derive reliable treatment decisions [1]. The technical evolution and the ubiquitous availability of ultrasonography equipment enables physicians nowadays to assess the supragenual and infragenual vessels with high resolution and accuracy before any invasive diagnostics while the known artifacts of non-imaging modalities remained the same (i.e., for ABI in diabetes). In this context, it is worth mentioning that 40 years ago the ABI was already measured after exercise and used for diagnosis of PAOD [10]. Duplex ultrasonography allows diagnosis of occlusions and estimation of the percentage of stenosis in addition to the Doppler ultrasonography method [11]. It sounds quite plausible that in PAOD the hemodynamics of the crural arteries, such as PSV, EDV, MD, TAMAX, should differ from those of healthy persons and this difference should be visible at rest; however, as our results showed (Table 1) this is not the case. Only the RI which reflects the pulse wave form (a value of 1 corresponds to a triphasic wave) showed a tendency to lower values (monophasic pulse wave form) due to proximal occlusions or stenosis. Obviously, healthy persons differ from PAOD patients more by the functional reserve than by the resting values due to the physiological autoregulation of the arterioles. Therefore, the healthy persons and the PAOD patients underwent a standardized exercise test. The ratios of the maximum/minimum parameter values after exercise and the resting values differed significantly between healthy persons and PAOD patients (Table 2). The amplitude of the autoregulation was much higher in healthy persons than in the latter. Therefore, we conclude that this is a much more reliable criterion to diagnose PAOD based on hemodynamics compared to resting values. When the patients would have been identified and treated according to guidelines, should the hemodynamics then return to “normal” values in order to indicate treatment success? From Table 1, it is obvious that only an elevation of the PSV of about 5 cm/s took place after treatment, which is clearly not a reliable change. The autoregulation did also not relevantly change after treatment as seen in Table 2 (and Fig. 1). Only the time course of the PI increased after treatment in PAOD patients as calculated by ANOVA (Table 2) but remained significantly different to the healthy values. Most parameters did not change at all shortly after treatment. Treatment success can be differently defined and in most cases would include the follow-up with documentation of event-free survival; however, it would be of value to foresee how long this would endure for the individual patient based on changes in hemodynamics immediately after treatment. The PI is thus the parameter that might serve for further evaluations of its event-free survival prediction. A cofactor which may influence the PI independently from the treatment state was the continuous proximal inflow of the crural vessel under investigation. This should be considered for interindividual comparisons.

A possible source of bias was postoperative complications (death and major amputation in two patients) which led to case-wise exclusions of postoperative data.

We can summarize that in order to reliably assess treatment effects by Duplex and Doppler ultrasonography, the patients should undergo an exercise test, the time course after exercise should be measured and compared to the pretreatment exercise test.
